# The oldest monofenestratan pterosaur from the Queso Rallado locality (Cañadón Asfalto Formation, Toarcian) of Chubut Province, Patagonia, Argentina

**DOI:** 10.1098/rsos.241238

**Published:** 2024-12-11

**Authors:** Alexandra E. Fernandes, Diego Pol, Oliver W. M. Rauhut

**Affiliations:** ^1^ SNSB, Bayerische Staatssammlung für Paläontologie und Geologie, Richard-Wagner-Straße 10, 80333 Munich, Germany; ^2^ Department of Earth and Environmental Sciences, Ludwig-Maximilians-University, Theresienstraße 41, 80333 Munich, Germany; ^3^ CONICET, Museo Argentino de Ciencias Naturales ‘Bernardino Rivadavia’, Avenida Patricias Argentinas 480, C1405 Ciudad Autónoma de Buenos Aires, Argentina; ^4^ GeoBioCenter, Ludwig-Maximilians Universität, Richard-Wagner-Straße 10, 80333 Munich, Germany

**Keywords:** Pterosauria, Monofenestrata, Jurassic, Toarcian, Patagonia, Argentina

## Abstract

As the first group of tetrapods to achieve powered flight, pterosaurs first appeared in the Late Triassic. They proliferated globally, and by the Late Jurassic through the Cretaceous, the majority of these taxa belonged to the clade Monofenestrata (which includes the well-known Pterodactyloidea as its major subclade), typified by their single undivided fenestra anterior to the orbit. Here, a new taxon *Melkamter pateko* gen. et sp. nov., represented by the specimen MPEF-PV 11530 (comprising a partial cranium and associated postcranial elements), is reported from the latest Early Jurassic (Toarcian) locality of Queso Rallado (Cañadón Asfalto Formation) and referred to the clade Monofenestrata, increasing our previously known taxonomic and geographic representations, and temporal range for this clade. This occurrence marks the oldest record of Monofenestrata globally and helps to shed critical light on the evolutionary processes undergone during the ‘non-pterodactyloid’-to-pterodactyloid transition within the Pterosauria. In addition, another single isolated tooth from the same locality shows ctenochasmatid affinities. These finds further elucidate the still-poor Gondwanan Jurassic pterosaur fossil record, underscoring that most of our current ideas about the timing and modes of pterosaur evolution during that period are largely based on (and biased by) the pterosaur fossil record of the Northern Hemisphere.

## 1. Introduction

Pterosaurs were the first clade of actively flying tetrapods and were highly successful during the Mesozoic, achieving a global distribution from the Triassic to the Cretaceous. Over that time, the pterosaur bauplan transitioned from the basal nonmonofenestratan ‘non-pterodactyloid’ body style to that of the more derived pterodactyloids [1–4]. This evolutionary event has become better understood in recent years, with the recognition of the clade Darwinoptera, which have been largely considered as ‘intermediate’ monofenestratans that show a morphological array of attributes during this transition, combining plesiomorphic characters of ‘non-pterodactyloids’ with pterodactyloid features [5,6]. Rather than exhibit intermediate character states, the evolutionary pathway of transitional pterosaurs often exhibited seemingly cherrypicked traits, adopting both ancestral and derived states simultaneously, which typifies mosaic evolution [6]. Within Monofenestrata (the clade comprised of Darwinoptera and Pterodactyloidea), members of Wukongopteridae (Darwinoptera to the exclusion of *Pterorhynchus*) exhibit characters from both basal non-pterodactyloids and derived pterodactyloids: a confluence of the nares and antorbital fenestra; a posteriorly inclined quadrate; a glenoid located on the scapula; reduced cervical ribs and a wing metacarpal about half the length of the first wing phalanx; still retaining an elongated tail enclosed by rod-like bony extensions made by the zygapophyses. Pterodactyloids then went on to adapt even more dramatically elongated crania and cervical vertebrae, a further reduction or loss of cervical ribs, elongation of the metacarpus, reduced tails, and a highly reduced or absent fifth toe. However, sound examples of these intermediate fossil forms are rare, particularly on the global scale, and therefore it has made challenging the process of identifying the mechanisms that underpin these transitions [6]. Although these changes are presumed to have had their evolutionary origins during the early–middle Jurassic time period, the evolutionary pathway towards pterodactyloids is still largely unknown [2]. Poor availability of geochronologic exposures from this time period, inadequate field sampling, and low bone preservation rates all hinder the understanding of this critical phase in which body plans changed so drastically [7,8].

The Mesozoic pterosaur record is abundant in the Northern Hemisphere, whereas the record in the Southern Hemisphere is comparatively more scarce [9–12]. With the possible exclusion of the Argentinian *Allkaruen koi* Codorniú *et al*. (2016) [13], non-pterodactyloid monofenestratan pterosaurs have thus far only been recovered from the Northern Hemisphere, namely the UK [5,14], Germany [15–17] and China (e.g. [6,18,19]), where they first appeared during the Bathonian [5,14]. The apparent success of these monofenestratan forms and their pterodactyloid descendants went on to supplant the rhamphorhynchid body style (which disappeared in the Early Cretaceous [4,20]), surviving through to the end-Cretaceous extinction. However, there is still a dearth of knowledge about non-pterodactyloid monofenestratans, specifically in terrestrial sedimentary settings [1,5,7,21,22].

Pterosaur records from Gondwana are especially meagre for the Jurassic [9,23], although Argentina itself contains fossil-yielding exposures from the Late Triassic to the Late Cretaceous (Toarcian–Coniacian), a span of over approx. 85 Ma [10,24]. Specimens have been recovered from at least 13 different fossil localities overall [25,13,23,26,27], but mainly come from two geographical regions: central -western Argentina (San Juan and San Luis Provinces) and Patagonia [13]. Most of the Jurassic records come from the highest parts of the Jurassic, with the exception of the latest Early Jurassic Cañadón Asfalto Formation [28]. This unit, which is exposed in the province of Chubut, comprises the bonebed containing *Alkaruen koi*, a Breviquartossan (the least inclusive clade containing *Rhamphorhynchus muensteri* Goldfuß [29] and *Quetzalcoatlus northropi* Lawson (1975) [30,31]), which is based on exceptional three-dimensionally preserved cranial and postcranial material [13,32]. Otherwise, only three other pterosaur taxa have been described from the Jurassic of South America thus far: *Herbstosaurus pigmaeus* Casamiquela (1975) [33], and *Wenupteryx uzi* Codorniú and Gasparini (2013) [34], from the Tithonian Vaca Muerta Formation of Neuquen, Argentina, and *Tucuadactylus luciae* Soto *et al*. (2021), from the latest Jurassic Tacuarembó Formation of Uruguay.

Here, new pterosaur fossils are presented from the locality of Queso Rallado of the Cañadón Asfalto Formation of Chubut Province, one of them representing a diagnosable new taxon, based on associated remains of preserved cranial and postcranial material. This specimen includes a partial cranium (including part of the premaxilla–maxilla–jugal–lacrimal–postorbital–squamosal–quadratojugal–quadrate complex) and a few postcranial remains (four vertebrae and a manual metacarpal), and its anatomy shows clear monofenestratan affinities. The second specimen is limited to an isolated tooth that presents derived similarities with ctenochasmatid pterodactyloids.

## 2. Geological and palaeontological setting

The Cañadón Asfalto Formation crops out in the northern central part of Chubut Province, Argentina. It is part of the sedimentary infill of the Cañadón Asfalto Basin, a large semigraben structure on central Patagonia that opened with the beginning of the South Atlantic in the Early Jurassic [35]. It belongs to the megasequence 1 of this infill, which represents the synrift phase of the basin, and conformably overlies the mainly volcanic Lonco Trapial Formation. The Cañadón Asfalto Formation is mainly composed of lacustrine shales, biogenic limestones and mudstones, with intercalated basaltic flows and pyroclastic deposits in the base, and fanglomerates and conglomerates in some sections [35–37]. The age of the formation has long been debated. Originally considered to be of Callovian-Oxfordian age (e.g. [38]), new biostratigraphic and radiometric dates have recently indicated an uppermost Early (Toarcian) to early-Middle Jurassic (Aalenian-Bajocian) age for this unit [39,40]. Even more recent radiometric dates, however, indicate that most (if not all) of the formation may be Toarcian in age [28,41,42].

**Figure 1.**
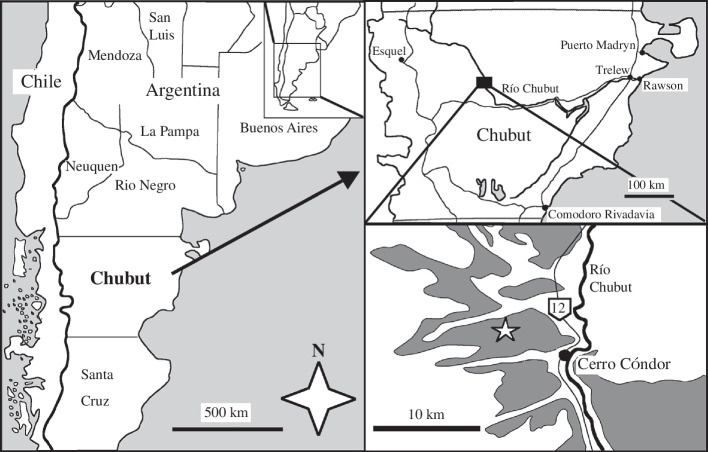
Map of the Queso Rallado locality (marked with a star) within the Chubut Province of Argentina, with the Cañadón Asfalto Formation shaded in dark grey (modified from [43]).

The Queso Rallado locality is located approx. 5.5 km northwest of the village of Cerro Cóndor (figure 1) in the area of the mid-course of the Río Chubut. The fossiliferous level is a 0.8 m thick carbonatic and partially silicified mudstone within the lower part of the section of the Cañadón Asfalto Formation. The exact stratigraphic correlation of the outcrops at Queso Rallado awaits publication, but a Toarcian age is considered the age of the fossiliferous level [40]. The fossiliferous strata at Queso Rallado have yielded a rich microvertebrate fauna of both aquatic and terrestrial animals. The fauna includes so far undescribed fishes, amphibians, crocodiles, pterosaurs and theropod teeth, as well as the turtle *Condorchelys antiqua* Sterli (2008) [44], the rhynchocephalian *Sphenocondor gracilis* Apesteguía, Gómez & Rougier (2012) [45], the heterodontosaurid ornithischian *Manidens condorensis* Pol, Rauhut & Becerra (2011), teeth of sauropod dinosaurs [46,47] and the mammaliaforms *Asfaltomylos patagonicus* Rauhut *et al*. (2003), *Condorodon spanios* Gaetano & Rougier (2012) [48], *Henosferus molus* Rougier *et al*. (2007) [49] and *Argentoconodon fariasorum* Gaetano & Rougier (2011) [50]. Other outcrops of the Cañadón Asfalto Formation have yielded the anuran *Nothobatrachus reigi* Báez & Nicoli (2008), the sauropod dinosaurs *Volkheimeria chubutensis* Bonaparte (1978) [38], *Patagosaurus fariasi* Bonaparte (1978) [38] and *Bagualia alba* Pol *et al*. (2020), the theropod dinosaurs *Piatnitzkysaurus floresi* Bonaparte (1978) [38], *Condorraptor currumili* Rauhut (2005) [51], *Eoabelisaurus mefi* Pol & Rauhut (2012) and *Asfaltovenator vialidadi* Rauhut & Pol (2019) [52], as well as the pterosaur *Allkaruen koi* Codorniú *et al*. (2016) [13].

## 3. Material and methods

The main specimen described herein comes from the microvertebrate locality of Queso Rallado [25]. It was found by mechanically breaking blocks that were recovered from the fossiliferous layer, during which the partial skull was split. Subsequently, the specimen was mechanically prepared, revealing additional bones originally still covered in matrix. Although the bones were not found in articulation, they were found in close association, and consist of a partial skull, four dorsal vertebrae, one long bone and two associated teeth. All measurements were taken with digital callipers. In addition, an isolated ctenochasmatid (?) tooth was recovered in the same locality (but from an unknown horizon), which is also reported upon. The specimens are housed at the Museo Paleontólogico ‘Egidio Feruglio’ (MPEF) in Trelew (Province of Chubut Province), Argentina under the collection numbers MPEF-PV 11530 a-c and MPEF-PV 2549[2012].

CT scans were performed at the three-dimensional Imaging Lab of the University of Tübingen, using a Nikon XT H 320 with a tungsten reflection target with a maximum voltage of 225 kV. This resulted in a stack of 2714 projections (voxel size = 0.113606). The data derived from the CT scan wereas segmented manually (Image Segmentation) with the software Avizo v9.2 (Thermo Fisher Scientific, MA, USA), using the brush function and interpolation, and rendered using the open-source software Blender. All CT files are available at https://www.morphosource.org/projects/000495959.

Phylogenetic analysis was conducted in TNT version 1.6 [53,54] using two matrices: the matrix of Fernandes *et al*. [55], which is in turn based on the matrix of Andres [56], and the matrix of Martin-Silverstone *et al*. [5]. In both matrices, we added the new taxon, checked the codings for *Allkaruen koi* (*Dimorphodon koi* in the matrix of Andres (2021) [56]; we changed the name back to *Allkaruen* in our matrix), based on our own observations, and added an additional character, the presence or absence of an ascending process of the maxilla. Thus, the final matrix based on Fernandes *et al*. [55] had 181 terminal taxa scored for 276 characters (51 continuous and 225 discrete; ordered and unordered), and the one modified from Martin-Silverstone *et al*. [5] had 70 terminal taxa scored for 136 characters (ordered and unordered). The data matrices are available in the electronic supplementary material, and also at http://morphobank.org/permalink/?P4589. Both matrices were analysed under equal and implied weights (with *k* = 12 [57]) using a traditional search with 2000 replicates, followed by TBR branch swapping on trees in memory. Reduced consensus methods were used to improve the resolution of the final results, using the pcrprune command [57,58].

## 4. Nomenclatural acts

The electronic edition of this article conforms to the requirements of the amended International Code of Zoological Nomenclature, and hence the new names contained herein are available under that Code from the electronic edition of this article. This published work and the nomenclatural acts it contains have been registered in ZooBank, the online registration system for the ICZN. The electronic edition of this work was published in a journal with an ISSN and has been archived and is available from the following digital repositories: PubMed Central and LOCKSS. LSID:

## 5. Results

### 5.1. Systematic palaeontology

**PTEROSAURIA** Owen, (1842) [59]

**MONOFENESTRATA** Lü *et al*. (2010) [6] *sensu* Andres *et al*. (2014) [1]

Genus ***Melkamter,* gen. nov**.

Type Sspecies ***Melkamter pateko*, sp. nov**.

#### 5.1.1. Etymology

Genus name ‘*Melkamter’* from the native Tehuelche word ‘mel’ meaning (in Spanish/English) ‘ala/wing’ and ‘kamter’ meaning ‘lagarto grande/big lizard’ (after the original translation of ‘pterosaur’ as ‘winged lizard’); the species epithet ‘*pateko*’ is derived from ‘pate’ meaning ‘rallado/rasped’ and ‘ko’ meaning ‘conjunto de huesos/set of bones’, an ode to both to the site of Queso Rallado, and the fractured preservational state of the fossil (translations from [60]).

#### 5.1.2. Holotype

MPEF-PV 11530 (Museo Paleontólogico Egidio Feruglio, Trelew, Argentina), consisting of at least a partial cranium (with counterslab) and two associated teeth, four dorsal vertebrae, one metacarpal (either I, II or III) and other bone fragments5.

#### 5.1.3. Type locality and hHorizon

Locality Queso Rallado, close to Cerro Cóndor, northern central Chubut Province, Argentina. Lower section of the Cañadón Asfalto Formation, latest Early Jurassic, Toarcian [28,40].

#### 5.1.4. Diagnosis

Non-pterodactyloid monofenestratan with a confluent naris and anteorbital fenestra and quadrate inclined at about 120°. Autapomorphies include: the presence of a small vestigial ascending process in the maxilla that does not reach the nasal or lacrimal dorsally; a maxillary body anterior to the vestigial ascending process that is higher than a posterior portion, with both portions divided by the vestigial ascending process delimited by a marked step; lacrimal and posterior processes of the jugal offset at about 55° angle. The taxon can furthermore be diagnosed by a combination of characters, including the marked dorsal deflection of the dorsal margin of the skull at the beginning of the nasoantorbital fenestra, resulting in a concave rather than straight outline of the dorsal skull margin in this region in lateral view, and the pointed anterior process of the quadratojugal that separates the posterior ends of the jugal and the maxilla.

## 6. Description

**Figure 2.**
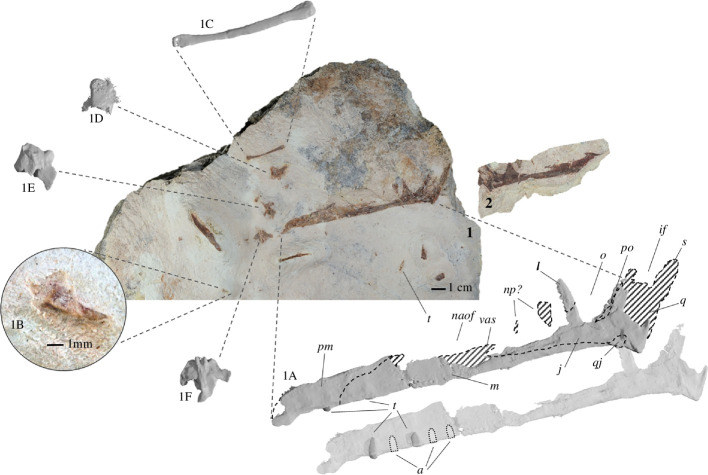
Photographs of MPEF-PV 115302, visible in medial views on the main block (1) with inset of the tooth (1B) and counterslab (2);. Rendered CT scan detail images (dashed areas represent visible bone that was too thin to be captured by CT scan) of the cranial fragment (1A), manual metacarpal (1C) and dorsal vertebrae (1D–F). *Abbreviations: a = alveoli; if = infratemporal fenestra; j = jugal; l = lacrimal; m = maxilla; o = orbit; naof = Nnasoantorbital fenestra; np = nasal process; pm = premaxilla; po = postorbital; q = quadrate; qj = quadratojugal; s = squamosal; t = tooth; vas = vestigial ascending process*.

MPEF-PV 11530 is preserved in a block (figure 2*a*) of a very thin and fine-grained pinkish-white to grey silicified mudstone, typical for the bone-bearing layers of the Queso Rallado locality, with one small partial counterpart of the cranial material (figure 2*b*). The elements that are preserved in the primary block include a partial cranium with two associated teeth, four dorsal vertebrae, and a metacarpal (either I, II or III). There are smaller bone shards and even a long bone shaft fragment interspersed throughout the remaining surface and interior (visible with CT scans) of the block,; however, they are of indeterminate morphology.

### 6.1. Cranium

**Figure 3.**
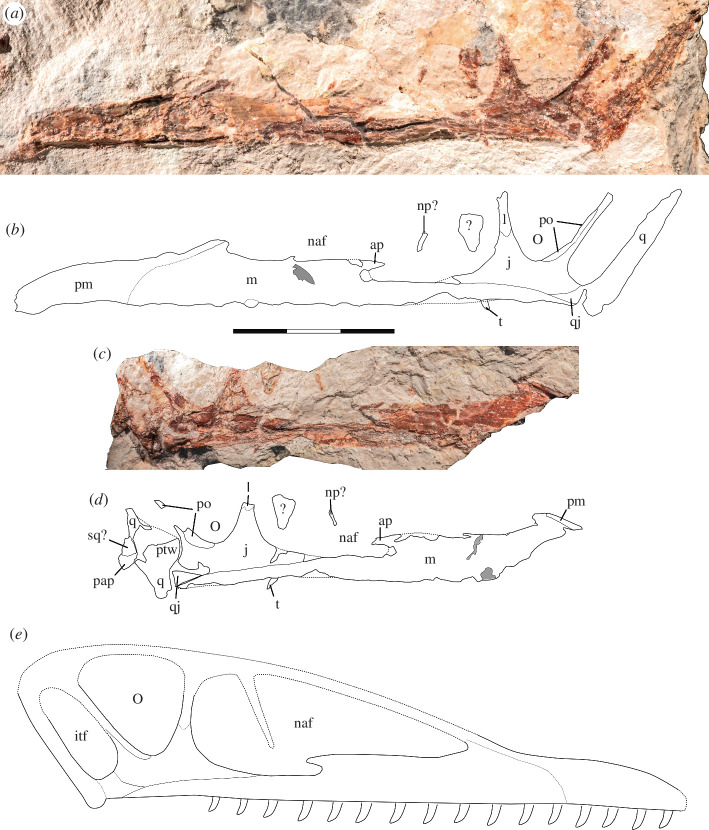
Holotype cranium of *Melkamter pateko* gen. et sp. nov., MPEF-PV 11530, in medial views. (*a*) pPhotograph of the main slab; (*b*) interpretative camera lucida drawing of the remains on the main slab; (*c*) photograph of the counterslab; (*d*) tentative interpretative camera lucida drawing of the remains on the counterslab; (*e*) reconstruction of the cranium (the size, number and arrangement of the teeth are conjectural). Scale bar is 3 cm. *Abbreviations: ap = ascending process of the maxilla; itf = infratemporal fenestra; j = jugal; l = lacrimal; m = maxilla; naf = nasoantorbital fenestra; np? = possible fragment of the ventral process of the nasal; o = orbit; pap = paroccipital process; pm = premaxilla; po = postorbital; ptw = pterygoid wing of the quadrate; q = quadrate; qj = quadratojugal; sq? = possible squamosal fragment; t = possible last tooth*.

The right side of the cranium is preserved and has been split between the main slab and the counterslab during the discovery of the specimen, so that the former mainly contains the outer bone layer, with much of the bone substance preserved on the counterslab. The cranium is anteroposteriorly complete, but missing the dorsal skull roof, occiput, lateral and posterior walls (laterosphenoids-orbitosphenoids), and the floor of the endocranial cavity. It is laterally crushed (complicating the identification of individual elements) but includes the regions of the premaxilla, maxilla, jugal, ventral parts of the lacrimal and postorbital, fragments of the squamosal, partial quadratojugal and quadrate (figures 2 and 3). The cranium has an overall length of approx. 131.3 mm from premaxillary tip to the dorsal posterior end of the quadrate (table 1), with the rostrum anterior to the anterior margin of the orbit measuring 94.8 mm (therefore comprising about 72% of the skull, whereas the condition in derived pterodactyloids usually occupies more than 80% [31,61–64]), and a nasoantorbital fenestra (*ca*. 51 mm) occupying about 39% of the anteroposterior skull length. The tip of the snout anterior to the nasoantorbital fenestra is approx. 39 mm long.

**Table 1.** Measurements of MPEF-PV 11530, in millimetres.

preserved cranium length (anteroposteriorly)	131.3 mm
cranium length to jaw articulation	112.1 mm
preserved maximum cranium height	24.9 mm
rostrum to anterior nasoantorbital fenestra	40.8 mm
cranium height at anterior nasoantorbital fenestra	12.7 mm
nasoantorbital fenestra length	51.6 mm
preserved posterior nasoantorbital height	20.4 mm
cranium length to nasoantorbital fenestra posterior margin	93.6 mm
mediodistal width of dental alveolus	1.7
preserved maximum orbit length	18.0
preserved maximum orbit height	15.8
infratemporal fenestra length	6.2
manual metacarpal length	29.2
manual metacarpal mid-width	1.3
dorsal vertebra centrum length	9.7
dorsal vertebra neural spine length	5.8

Of the main cranial openings, the ventral parts of the nasoantorbital fenestra, the orbit and the infratemporal fenestra are visible. The nares and anteorbital fenestra are visibly confluent to form a nasoantorbital fenestra, with a pointed anterior margin, and a ventral margin that is placed on a more dorsal level in the anterior half (probably corresponding to the original nares) than in the posterior half (probably corresponding to the original antorbital fenestra). The two portions of the ventral margin are separated by a marked step that is slightly undercut from the posterior side, and which we interpret as a vestigial ascending process of the maxilla (see below). Above this process, the openings are confluent and there is no indication of a dorsal extension of the ascending process contacting either the nasal or the lacrimal dorsally (cortical bone striations on the process also markedly run solely in the anteroposterior plane).

A platy, triangular bone shard is dorsally placed within the posteriormost part of the nasoantorbital fenestra, with no contact made to any other bone. This might represent the free nasal process that intrudes into the nasoantorbital fenestra in many basal monofenestratans, although the bone seems to be too broad and bulky, and very posteriorly placed if compared with other taxa (e.g. [6,65]). It could also be the displaced anterior–dorsal region of the lacrimal or the nasal process of the frontal. Alternatively, a small bone shard more anteriorly within the nasoantorbital fenestra in between the triangular fragment and the end of the ascending process of the maxilla could also potentially represent this process. This fragment is thin, almost vertically and very slightly anteroventrally inclined and flexes posteriorly towards its end. In comparison with other monofenestratans, its ventral end would be relatively closer to the ventral margin of the nasoantorbital fenestra, but this might not be unexpected in a very early representative of this clade. However, as there are other bone fragments distributed all over the slab, none of these two shards can be identified as the free nasal process with any certainty.

The orbit has straight anterior and posterior borders that diverge throughout the preserved height of the opening so that its anteroposterior width increases dorsally. The entire shape of the orbit cannot be established, due to the missing dorsal region. However, what is preserved does appear pyriform, with a rounded and approaching Vv-shaped ventral margin. The orbit measures 20.3 mm at the widest preserved anteroposterior point. The jugal–lacrimal strut separating the orbit from the nasoantorbital fenestra is triangular, with a wide ventral base and its long axis almost perpendicular to the alveolar border, as in darwinopterans (e.g. [6,66–68]) and basal pterodactyloids (e.g. [65]), but unlike the strongly anteriorly inclined strut in many non-monofenestratan pterosaurs, such as *Rhamphorhynchus* and *Scaphognathus* [65], *Dorygnathus* [69–71], *Campylognathoides* [15] or *Dimorphodon* [69]. The infratemporal fenestra was apparently high, but anteroposteriorly narrow and anteroventrally inclined so that its ventral end was placed below the orbit, as in many pterosaurs.

Although the crushed state of the specimen makes it difficult to discern any suturing (also further complicated by the sporadic distribution of cortical bone on either the part or counterpart of the fossil), it appears that the premaxilla is short, and is potentially fused to the maxilla where a slight dorsoventral crack occurs at about 20 mm from the anteriormost tip. A thin posterior dorsal process of the premaxilla seems to extend from here posterodorsally, flanking the dorsal margin of the maxilla and probably forming the dorsal margin of the nasoantorbital fenestra, as in other pterosaurs. There is no sign of a premaxillary crest, although one could have been more posteriorly placed along the dorsal margin of the skull than is preserved on the specimen.

The maxilla extends posteriorly from the contact with the premaxilla, contributing to the anterior and ventral margins of the nasoantorbital fenestra, until it is overlapped dorsally by the anterior process of the jugal. The maxilla continues below the jugal to at least the mid-length of the orbit. The anterior half of the maxillary body below the nasoantorbital fenestra is approximately twice as high dorsoventrally (*ca*. 8 mm) than the posterior half (maximally 4 mm). In between these two sections, a small incision into the posterior end of the anterior half is present within the nasoantorbital opening. We interpret this incision as marking the posterior end of a vestigial ascending process of the maxilla, which is thus almost entirely posteriorly directed and does not contact a ventral process of the nasal or lacrimal (figures 3 and 5*b*). The dorsal margin of this process is partially preserved in the counterslab, and curves downwards in its posterior part, resulting in a very slender termination of the process (figure 5).

**Figure 4.**
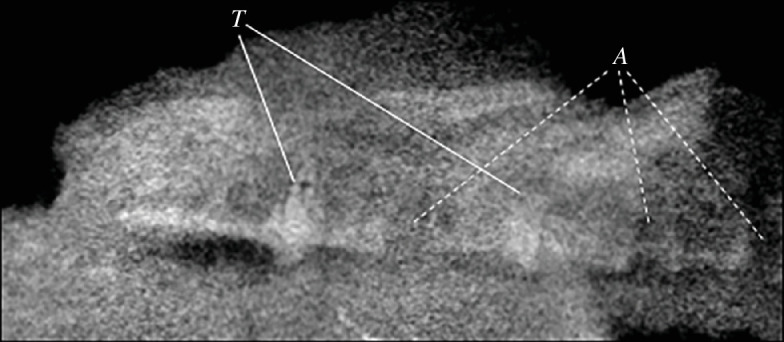
CT scan showing the position of two teeth (T*t*) scanned on the slab inside the maxilla, with three additional empty alveolar (A*a*) sockets.

**Figure 5.**
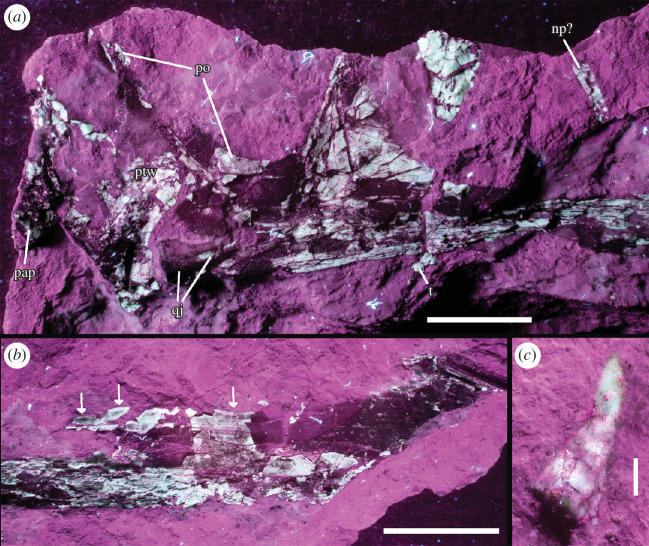
Details of the holotype cranium of *Melkamter pateko* gen. et sp. nov., MPEF-PV 11530, under UV light. (*a*) pPosterior part of cranium as preserved on the counterslab; (*b*) anterior part of the maxilla as preserved on the counterslab; (*c*) isolated tooth, preserved on the main slab. Abbreviations as in figure 3. Arrows in (*b*) point to preserved original margins of the base and the distal part of the ascending process of the maxilla. Scale bars, are 1 cm(*a, b*) 1 cm and 1 mm (*c*) 1 mm.

One tooth is visibly well-preserved on the surface of the block (figure 2*b*) as well as one additional fragmentary tooth, both in close association to the cranium, and although no teeth are visibly attached to the rostrum, CT data (figures 2 and 4) show that several alveoli (at least 5 per side) are indeed present and regularly spaced throughout the maxillopremaxillary region, with at least two partial teeth present within their individual alveolar sockets. Several empty sockets are also visible, but image quality precludes discerning how far anteriorly or posteriorly this toothrow extends. There is a small, posteroventrally inclined splinter in the posterior part of the maxilla that might represent a remnant of a tooth; if correctly identified, the tooth row would at least extend to the posterior margin of the nasoantorbital fenestra. Individual alveoli are widely spaced, with the distance between alveoli equalling almost two tooth widths in the anterior part of the maxilla. The completely preserved tooth (figure 5*c*) is apicobasally short (4.3 mm), and anteroposteriorly thin (1.7 mm at the widest point of the base), with the shape of a curved cone, similar to that of basal pterodactyloids such as *Germanodactylus* sp. and *Pterodactylus* sp. The tooth also exhibits a gradual apical taper with a gentle curvature, such that the tip is at an about an 10° offset to its base.

The triradiate jugal tapers to a point anteriorly and contacts the maxilla anteriorly with a seemingly elongate process, together forming the flat ventral margin of the nasoantorbital fenestra (figure 3). Although the exact contact of the anteroventral jugal process with the maxilla cannot be confidently distinguished, it can be estimated to form approx. 13 mm of the posterior ventral margin of the nasoantorbital fenestra. The lacrimal process of the jugal rises in an almost perpendicular curve in respect to its ventral margin. Both this ascending process and the posterior postorbital ramus taper dorsally, reaching about the halfway point of the preserved orbit. The lacrimal process is considerably more massive than the postorbital process and contacts the ventral ramus of the lacrimal at about the mid-height of the orbit, or slightly lower. Although the suture is poorly preserved, the ventral end of the lacrimal seems to slot into a notch in the dorsal end of the jugal. The postorbital process is slender and reaches approximately as far dorsally as the lacrimal process (figure 5). Anteriorly, it is overlapped by a rather massive ventral process of the postorbital, which forms the entire posterior margin of the orbit and reaches the mid-length of this opening ventrally. The angle formed by the lacrimal and postorbital processes of the jugal is set at about 55°. A short and dorsoventrally broad posterior process of the jugal is also present. Only the anterior end of the quadratojugal is preserved. It is triangular in outline, tapering anteriorly, and inserts in between the ventral margin of the posterior end of the jugal and the dorsal margin of the posterior end of the maxilla, as in *Cycnorhamphus* [72].

The quadrate appears long and broad, inclined posterodorsally backwards at about 126° from the plane of the palate. It forms the posterior boundary of the infratemporal fenestra. On its ventral-most point, there is a small, bulbous articular protuberance for articulation with the mandible. The large, anteriorly rounded pterygoid wing of the quadrate is partially preserved. It is offset from the ventral end of the quadrate and rapidly expands anteriorly in its ventral part, whereas the dorsal end grades more gradually into the quadrate shaft. On the counterslab, the end of the paroccipital process is preserved, and anterodorsal to it is a strongly eroded piece of bone that most probably represents a remnant of the squamosal.

**Figure 6.**
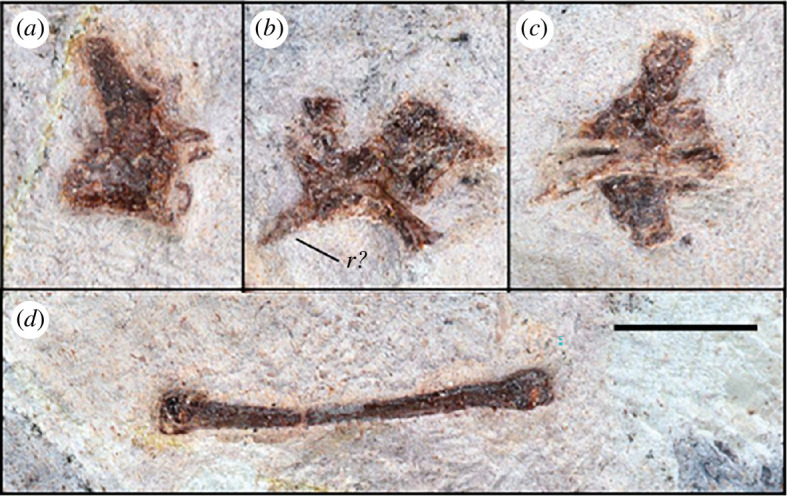
Details of MPEF-PV 11530 postcranial material: (*a–c*) dorsal vertebrae and (*d*) manual metacarpal. Scale bar is 1 cm. *Abbreviations: r = rib*.

Four dorsal vertebrae are preserved in the block (two isolated, and two in close articulation with each other) (figure 6*a–c*). Of the two isolated vertebrae preserved, one is visible in lateral view (figure 6*a*) and one in dorsal view (figure 6*c*), with the articulated vertebrae in anterolateral view (figure 6*b*). From these perspectives, the shapes of the centra are procoelous, and in lateral view, there is a slight dorsoventral constriction of each centrum, giving a spool-shaped profile. The transverse processes have an anteroposteriorly broad base until the capitular facet is reached, from which point they project dorsolaterally as they taper gently to a rounded tip (figure 6*a*). The location of the capitular facet (about halfway along the length of the transverse process) also contributes anteriorly to the lateral margin of the prezygapophysis, similar to darwinopterans [9]. Due to the taphonomic state of the vertebrae, it is not possible to distinguish the presence of any foramina. The neural spines of the two articulated dorsals contact each other completely, with only a very faint line visibly demarcating them. These two dorsals also potentially preserve an articulated fragmentary dorsal rib, although this is difficult to distinguish in their fractured state.

One metacarpal is completely preserved (either metacarpal I, II or III). A shaft fragment (potentially another metacarpal I–III) and another large indeterminate bone shard are also preserved on the surface of the block. However, all are too eroded to glean any further information.

### 6.2. Systematic palaeontology

**PTEROSAURIA** Owen, 1842 [59]

**PTERODACTYLOIDEA** Plieninger, 1901 [73]

**CTENOCHASMATIDAE(?) *indet.*** Nopsca, 1928 [74] *sensu* Unwin, 2003 [31]

**Figure 7.**
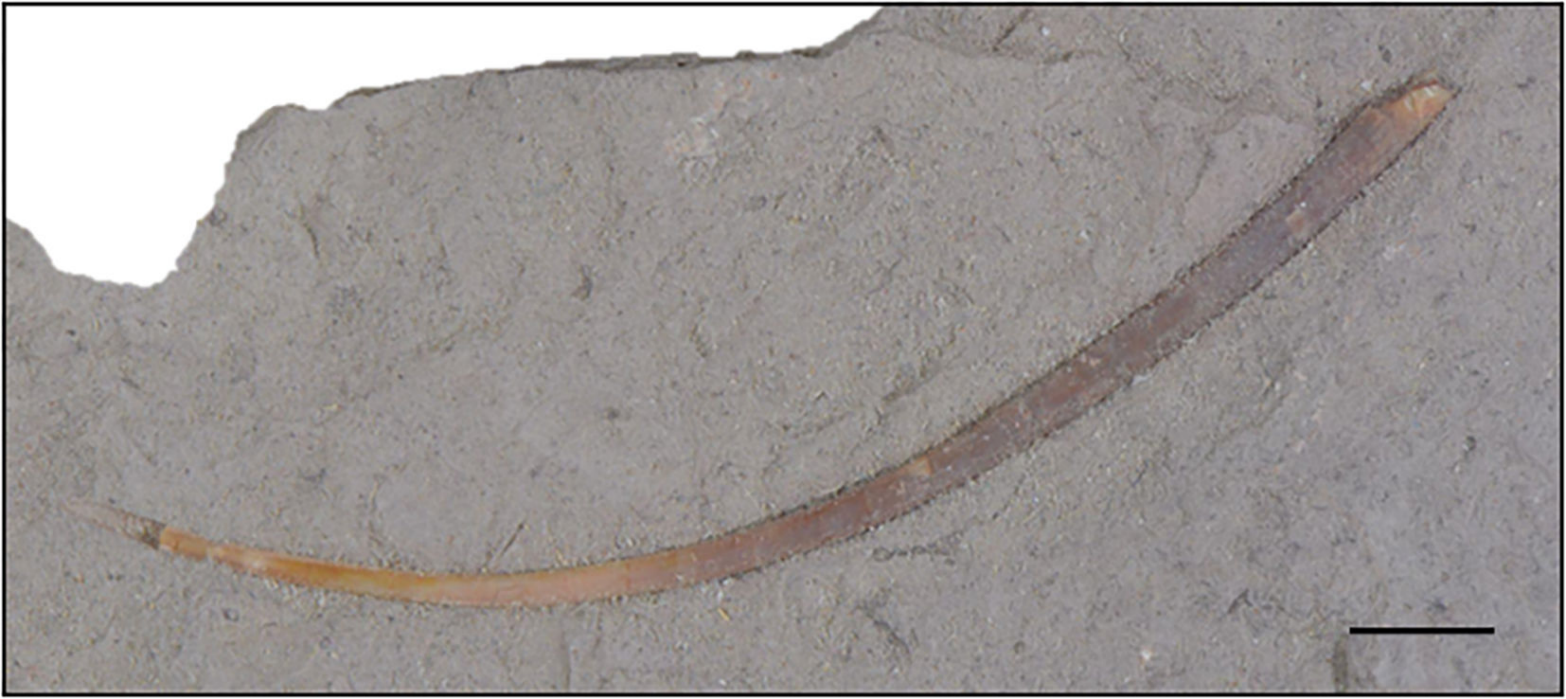
Isolated ctenochasmatid(?) tooth MPEF-PV 2549[2012], also from the Queso Rallado locality. Scale bar is 1 cm.

In addition to the above-mentioned specimen, a separate block containing an isolated tooth was also recovered from the Queso Rallado locality of the Cañadón Asfalto Formation, which closely resembles the teeth of ctenochasmatids (figure 7). The sediment comprising the block is different in colour and texture from the above-mentioned specimen, likely indicating that it originated from a different horizon. The tooth is in good condition overall, preserving most of the crown, and missing only the apical-most tip (although a natural mould in the surrounding sediment retains its overall original shape). The base of the tooth appears to have been broken (likely near the crown–-root boundary), perhaps during feeding, but the lack of erosion over the entire tooth surface indicates that it was not likely to be much transported after its dissociation from the rostrum. The tooth is remarkably elongated, slightly recurved, and measuring 47 mm from base to tip, 1.5 mm wide at its base and gradually tapering to 0.5 mm wide at its apical tip. At the base, it is lenticular in cross-section, but becomes more circular in cross-section as it approaches the apex, as in most ctenochasmatids, and similar to the morphology of the anterior teeth (from the third to the sixth–-seventh) of *Pterodaustro guinazui* [24]. There is an ombre-like colour change from base to tip, varying from grey to beige, which is also indicative of the dentine-to-enamel change, with the enamel thickening apically (the lower grey 30 mm of the tooth representing exposed dentine as it is less shiny than the more apical beige enamelled region). The enamel is completely smooth and lacks any ornamentation. Due to the proportion of dentine-to-enamel present, it is likely that the tooth is from the anteriormost region of a ctenochasmatid rostrum (as anterior teeth are also subject to more breakage during feeding) [75,76].

### 6.3. Phylogenetic analysis

**Figure 8.**
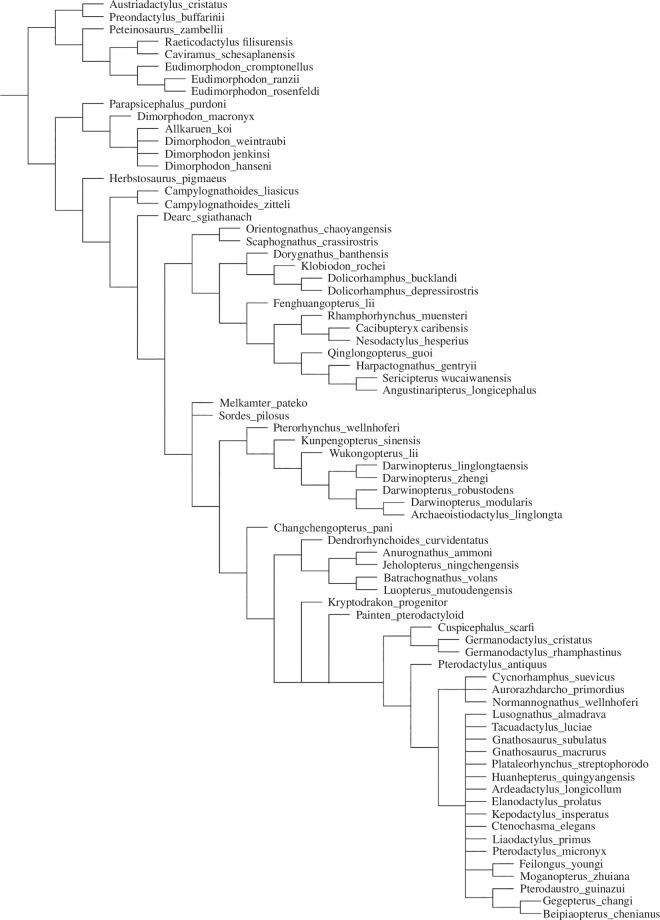
The relationship of *Melkamter pateko* to the Pterosauria, using the Fernandes *et al*. [55] matrix (simplified in Adobe Illustrator).

The analysis of the matrix based on Fernandes *et al*. [55] under equal weights resulted in the recovery of 12 equally parsimonious trees with a score of 1388.014 (figure 8). The results are generally comparable to those published by Andres [56], but show less resolution, with some polytomies being present. *Melkamter* is found in a polytomy with *Sordes* just outside the Darwinoptera–-Pterodactyloidea clade in this analysis. Interestingly, *Allkaruen* is still found in a polytomy with species of *Dimorphodon* in the Dimorphodontia, as in the original analysis of Andres [56], despite the revised scorings for this taxon. Constraining *Allkaruen* into the clade including *Sordes* and monofenestratans (as found by [5,13]) requires three additional steps (score of 1391.221) and finds this taxon within derived pterodactyloids as a member of the Istiodactylinae; given that the holotype of *Allkaruen* mainly consists of a complete, three-dimensionally preserved braincase [13], the rather limited sampling of braincase characters might account for these vastly differing results.

using the Fernandes *et al*. [] matrix (simplified in Adobe Illustrator).

**Figure 9.**
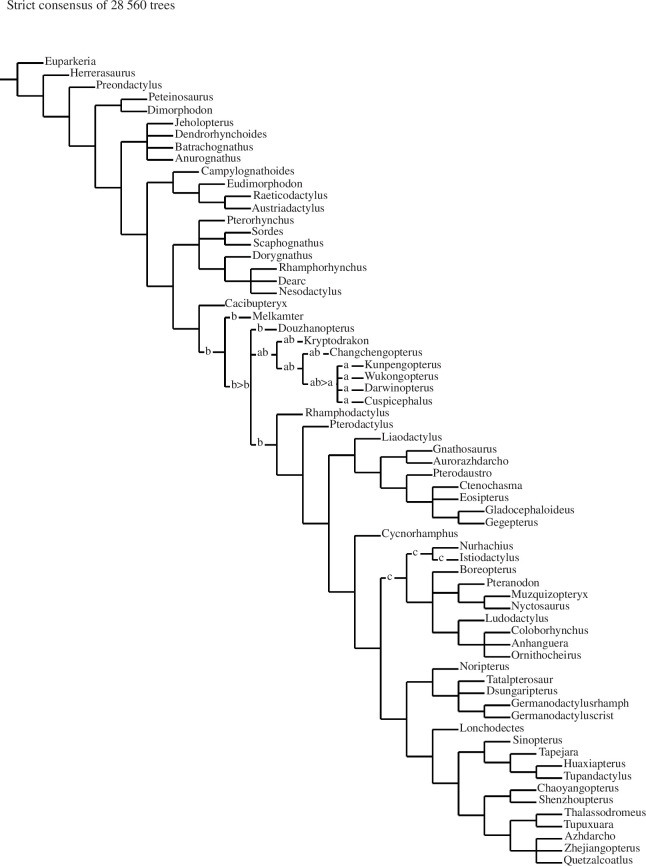
The relationship of *Melkamter pateko* to the Pterosauria, using the Martin-Silverstone *et al*. [5] matrix (reduced consensus; strict consensus in electronic supplementary material).

The equal weights analysis using the matrix based on Martin-Silverstone *et al*. [5] recovered 28  560 equally parsimonious trees with a length of 556 steps (figure 9). The strict consensus of these trees largely confirms to the results of Martin-Silverstone *et al*. [5], but shows both *Melkamter* and *Allkaruen* in a polytomy with darwinopterans at the base of Monofenstrata. Reduced consensus methods identify *Allkaruen* as a problematic taxon in this part of the tree, the *a posteriori* deletion of which results in *Melkamter* being found as the earliest branching monofenestratan, followed by a monophyletic Darwinoptera and pterodactyloids. *Allkaruen* can take multiple positions within this phylogenetic hypothesis, either below or just above *Melkamter*, as a sister taxon to the latter, as a darwinopteran or as the earliest branching pterodactyloid. The implied weights analysis of this matrix recovered 51 trees of a score of 22.36738 and finds *Melkamter* and *Allkaruen* in a polytomy at the base of Monofenestrata.

## 7. Discussion

### 7.1. Phylogenetic position and evolutionary implications

The phylogenetic analyses based on both different datasets agree in placing the new taxon described here just outside the clade encompassing darwinopterans and pterodactyloids (for the [5] phylogeny) or darwinopterans and pterodactyliformes (for the [55] phylogeny). In the implied weights analysis of the [55] matrix, *Melkamter* is in a polytomy with *Sordes*, as oldest and earliest branching monofenestratan, followed by the Darwinoptera and Pterodactyliformes (*sensu* [56]). As the Monofenestrata are an apomorphy defined clade, characterized by the presence of a confluent nasoantorbital fenestra [56], the presence of this trait in the material described here and its phylogenetic position allows its reference to this clade. The presence of *Melkamter* in the Cañadón Asfalto Formation therefore marks the earliest worldwide occurrence (late Early Jurassic, Toarcian [28,40]) of a monofenestratan pterosaur, predating the currently oldest member of that clade [5] by at least 8 and probably 10 million years. However, the clade had seemingly achieved a wider distribution by at least the Middle Jurassic [5], and a probably worldwide distribution by the early Late Jurassic.

*Melkamter* is the first and only non-pterodactyloid monofenestratan from Gondwana. However, direct comparisons of *Melkamter pateko* with other non-pterodactyloid monofenestratans (outside of the darwinopterans) is complicated for lack of their overlapping anatomical elements. In particular in comparison with its fellow Argentine taxon, *Allkaruen koi* from the same formation, it therefore cannot be entirely excluded that they are the same taxon as the two taxa do not have any overlapping material in common. However, in additional to the results of the phylogenetic analyses of the [55] matrix, where these two taxa come out in widely different positions (and in which forcing together *Melkamter* and *Allkaruen* requires five additional steps), the only morphological evidence that is comparable, the relative size and shape of the alveoli, also contradicts this, as the alveoli of the similar-sized holotype of *Allkaruen* seem to be relatively larger and more mediolaterally compressed than those of *Melkamter*.

**Figure 10.**
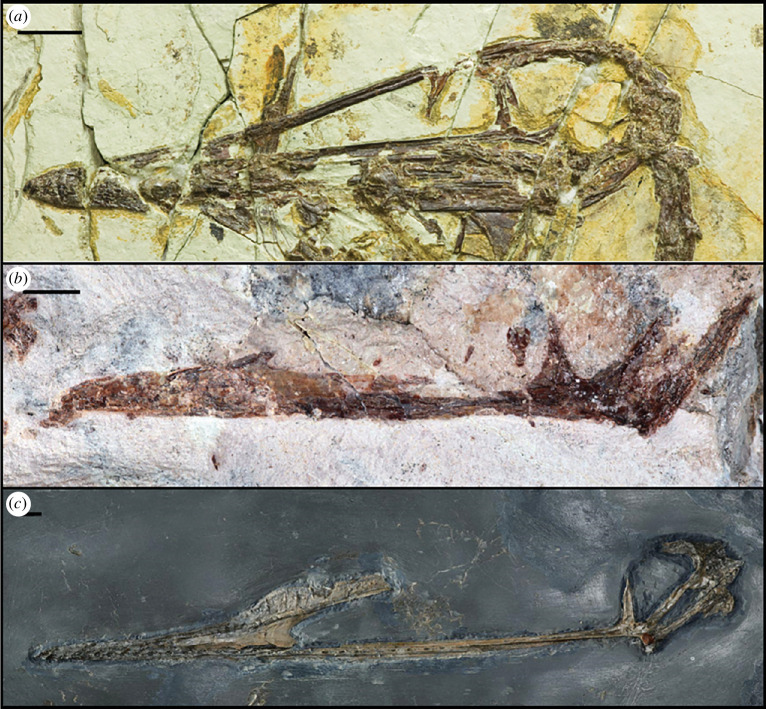
Comparison of (*a*) *Darwinopterus linglongensis* (IVPP V16049; mirror-imaged for comparison), (*b*) *Melkamter pateko*, and the newly- prepared left side of (*c*) *Cuspicephalus scarfi*. Scale bars are 1 cm.

Regarding comparison with darwinopterans, since the time of its initial description, *Cuspicephalus scarfi* Martill & Etches (2012) [77] (figure 10*c*) has been further prepared from its opposite side (which is better preserved), allowing for a more direct observation of its morphology and more direct comparison with *Melkamter pateko* and other darwinopterans (figure 10). Although the cranium of *Cuspicephalus* is about twice the length of that of *Melkamter*, they both exhibit broad ascending processes of their jugals, as do *Kunpengopterus sinensis* and *Darwinopterus modularis* (but unlike *Darwinopterus linglongensis*). However, although some phylogenies do indeed regard it as a darwinopteran [77,78,5], one phylogenetic analysis herein recovers *Cuspicephalus* as a germanodactylid [55], instead of its initial assignment as a non-pterodactyloid monofenestratans [77].

The overall shape of the cranium in *Melkamter pateko* suggests a high skull, as in *Darwinopterus linglongtaensis* Wang et al. (2010), [79], which is higher than that of both *Kunpengopterus sinensis*[79] and *Darwinopterus modularis*[79]. *Kunpengopterus* lacks a premaxillary crest (tentatively matching the condition of *Melkamter*), making it unlike other darwinopterans. However, the quadrate of *Kunpengopterus* is inclined more dramatically than in the specimen herein (150°, as opposed to about 126° in *Melkamter*). The inclination found in *Dimorphodon* is 95° [80,81], in *Parapsicephalus* it is between 115° and 130°, *Eudimorphodon* and *Scaphognathus* are at 120° [82,83], *Dorygnathus* and *Campylognathoides* are between 120° and 130° [70,71], *Rhamphorhynchus* is at 130°– 150° [61,65,84] and *Angustinaripterus* is at 140° [85]. Among darwinopterans the angle varies from about 130° to 132° in *Darwinopterus linglongtaensis* to 142° in *Kunpengopterus*, and 140° in *Cuspicaphalus* [77]. Germanodactylids are further set at about 148°, with more derived pterodactyloids having even larger angles of inclination. This places *Melkamter* as having a more acute angle than the darwinopterans (figure 10), with a value closer to those found in the more basal scaphognathines and campylognathoidids than in the more derived pterodactyloids.

Although potentially ontogenetically variable, the angle of the orbit (formed by the lacrimal and posterior processes of the jugal, and measured from photographs of each specimen) in *Cuspicephalus* is set at 45°, *Parapsicephalus* at 45°, *Angustinaripteus* at 46°, *Tupandactylus* at 45°, *Dimorphodon* at 47°, *Darwinopterus modularis* at 60°, *Germanodactylus cristatus at* 63°, *Pteranodon* at 65°, *Germanodactylus rhamphastinus at* 70°, *Dorygnathus* at 72°−96°, *Pterodactylus micronyx* (the neotype) at 75°, *Cycnorhamphus at* 76°, C*ampylognathoides* at 77°, *Eudimorphodon* at 80°, *Ctenochasma* at 80°−85°, *Scaphognathus* at 80°−100°, *Rhamphorhynchus* at 82°, and 90° in *Pterodactylus antiquus* (the holotype) [65,70–72,84–90]. *Melkamter* expresses an angle of about 55°, which places it with a value closer to those found between *Dimorphodon* and *Darwinopterus*.

Darwinopterans usually exhibit pterodactyloid-like elongated crania with confluent nasoantorbital fenestrae, free nasal processes, inclined quadrates and short peg-like teeth [91,6,79,92–94], all of which are exhibited by *Melkamter*. Despite their traditionally held modular evolution, however, it has also been recently argued that darwinopterans should no longer be considered a directly transitional group between basal non-pterodactyloids and the Pterodactyloidea, but rather a sister group to the Pterodactyloidea, along with the scaphognathines and rhamphorhynchines [5]. One significant autapomorphy seen in *Melkamter* holds merit in making this distinction: that of a vestigial ascending process of the maxilla, which is absent in all other known monofenestratans, and could potentially show the evolutionary pathway to the pterodactyloids losing this feature completely.

Concerning the tooth MPEV PV 2549, a secure identification of such an isolated element is, of course, difficult. The extreme elongation of this tooth is remarkable; such elongate teeth are unknown from any other terrestrial Mesozoic vertebrate clade, but elongate, recurved and pointed teeth are quite frequently found in pterosaurs [(see [4,62]). A further argument for a pterosaur identification of this tooth is the unusual distribution of enamel: an apical enamel cap and dentine base to the crown (with a distinct enamel–-dentine junction demarcating them), which is similarly found in other pterosaur clades (e.g. [4,75]). Inclusion of the tooth in the phylogenetic analyses above was attempted, but results were inconclusive for lack of characters. However, within pterosaurs, the tooth is most similar to the teeth of ctenochasmatids, which have strongly very elongate, slender teeth with a marked curvature in at least the mesial teeth (see e.g. [87]). If the presence of a (probably early branching) ctenochasmatid from the Cañadón Asfalto Formation can be confirmed, this would considerably extend the fossil record of pterodactyloids and place the origin of this clade firmly in the Early Jurassic. The tooth is unusually large for known ctenochasmatids, being approximately double the length of the mesial teeth of a skull of *Gnathosaurus* from the Tithonian Altmühltal Formation of Eichstätt, Germany [87] and even larger if compared to known large specimens of ctenochasmatids (e.g. [95]) but the discovery of the very large jaw of *Lusognathus* demonstrates that ctenochasmatids could reach large sizes already in the Jurassic (i.e. the estimated 3.6 m wingspan of *Lusognathus almadrava* [55]. Therefore, the tooth is here tentatively assigned to the Ctenochasmatidae, thus extending the stratigraphic range of this clade by at least 10–15 million years [92]. As ctenochasmatids are well nested within Pterodactyloidea, this would furthermore extend the entire clade and its early diversification into the Early Jurassic. However, as other, more basal pterosaurs also show elongate and curved teeth, including the rhamphorhynchids (e.g. [65,70,71]), it cannot be excluded that this tooth represents an independent acquisition of such extremely elongate teeth, allowing for the possibility that it could belong to some other novel taxon entirely. Nevertheless, the tooth represents a taxon different from both *Allkaruen* and *Melkamter*, thus further increasing pterosaur diversity in the Cañadón Asfalto Formation.

### 7.2. Dentition and ecological implications

Great variability is shown in the basal monofenestratan dentition. *Propterodactylus frankerlae* Spindler *et al*. (2024) [16] has pointed, conical long teeth with large interdental spaces and rostrolaterally directed fangs in the rostral jaw area [15,16], *Darwinopterus modularis* exhibits spike-like teeth [3], *Darwinopterus linglongtaensis* and *Kunpengopterus sinensis* have short blunted cone-shaped teeth, *Wukongopterus lii* has cone-shaped and very pointed teeth [79], and *Darwinopterus robustadens* exhibits relatively stout teeth [3,67]. This variation in dental morphology has been interpreted as evidence of niche partitioning [3,18,67,79] and the feeding on different prey items, although all these taxa are overall generally regarded as being insectivorous or piscivorous [18,94]. Based on the short, stout, conical tooth morphology, darwinopterans were probably insectivores [18,94], but the high tooth count of *Cuspicephalus scarfi* could also potentially indicate piscivory [77,91]. This is all in keeping with the relative abundance of arthropods present in some concurrent deposits (e.g. [18]), and fish in others (e.g. the Tiaojishan Formation and Daohugou beds of [79]). The dental morphology and regular tooth spacing of the *Melkamter* appear to be of the insectivorous morphotype [94], warranting more similarity with wukongopterids, which also exhibit interalveolar spacing that is greater than their tooth lengths [22], characteristics which are shared with the specimen herein. Whereas many pterosaurs from the ‘classic’ marine Lagerstätten (e.g. the Rhamphorhynchidae) show adaptations to a piscivorous diet (see [62,94]), this concentration of insectivorous forms in the early branching monofenestratans might support the suggestion of Andres *et al*. [1] that the origin of pterodactyloids might be found in truly terrestrial rather than nearshore or insular environments. This is especially the case in the phylogenetic hypothesis based on the Andres [56] and Fernandes *et al*. [55] datasets, in which the Anurognathidae, for which there is the strongest evidence and consensus for an insectivorous diet [94], represent the immediate outgroup to Pterodactyloidea. Given the otherwise expressed dietary plasticity in pterosaurs (see [94,96]), such a preference for terrestrial prey in non-pterodactyloid monofenestratans could be unexpected if these animals lived in nearshore environments or on islands in a lagoonal setting.

Ecological requirements (such as prey preferences) do affect the dispersion of animals, even beyond their ability to fly [97]. However, considering that the prey of insectivorous animals also fly (albeit with a likely smaller range), this could potentially indicate that insectivorous monofenestratans could have a wide dispersion (even across wider geographical barriers than those feeding solely on lacustrine fish, for example), setting different natural geospatial limits on the ecological niches that they occupied. Thus, in a still Pangean world during the Early and Middle Jurassic, a largely insectivorous diet might have represented an important adaptation in the success of basal monofenestratans and their descendants, the pterodactyloids.

## 8. Conclusion

**Figure 11.**
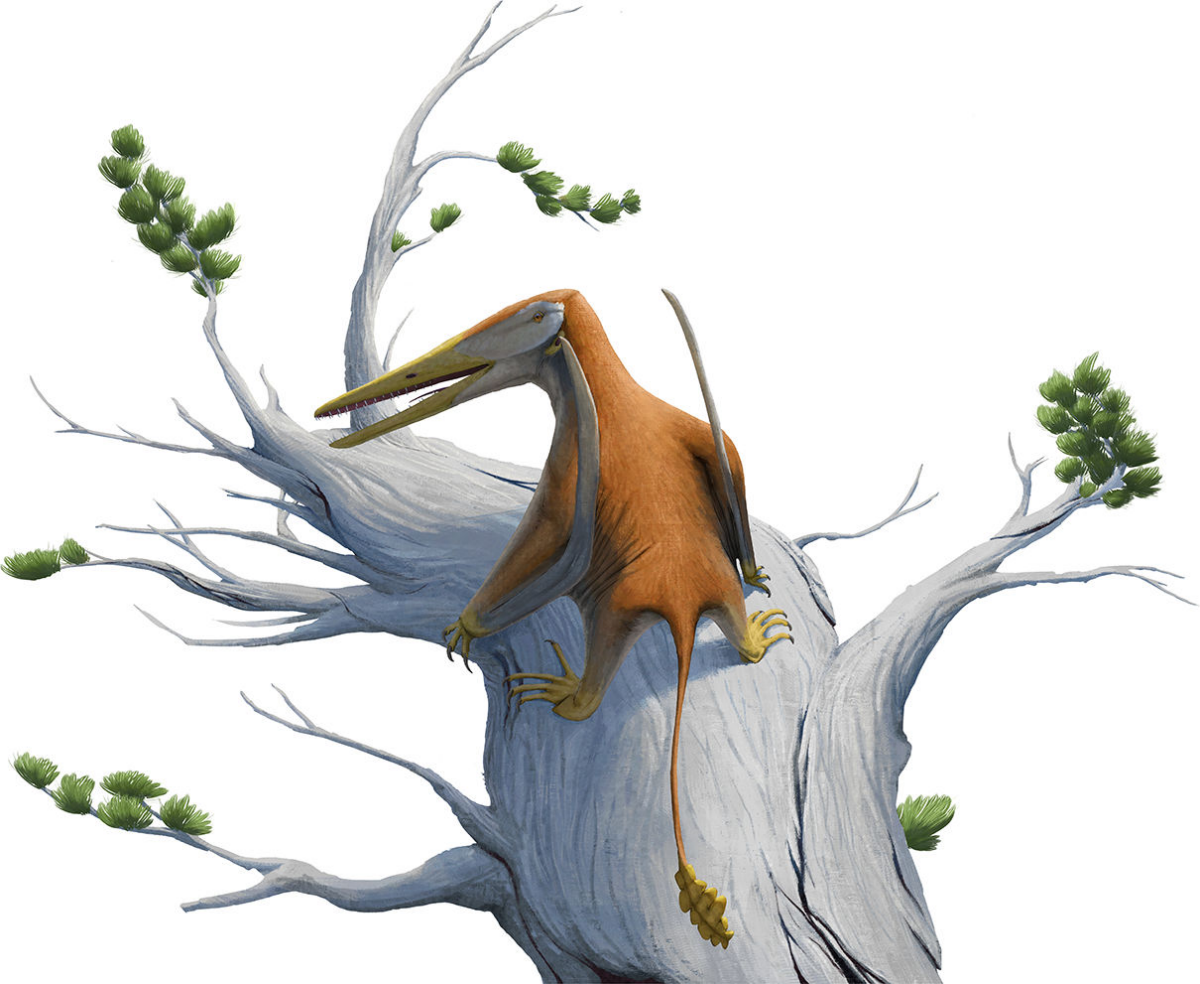
Artistic reconstruction of *Melkamter pateko* by Pedro Andrade.

It has long been suspected that pterodactyloids were already present by the end of the Early Jurassic [14,21], and that pterosaurs had already reached relatively high levels of taxonomic and morphological diversity by the Middle Jurassic [5], although the dearth of available fossil material has made these claims difficult to concretely establish. *Melkamter pateko* (figure 11), from the latest Early Jurassic of Chubut Province, represents the so far most conclusive evidence for the presence of Monofenestrata during the late Early Jurassic, while also contributing to their morphological diversity with the novel traits expressed in this new taxon. Furthermore, if confirmed by future finds, the possible presence of a ctenochasmatid, currently indicated by a single tooth, would not only place the origin of pterodactyloids into the Early Jurassic, but even indicate that their initial diversification already happened during that time. Although the Lagerstätten of the Northern Hemisphere have traditionally dominated in our understanding of the diversity and dispersion of pterosaurs over time, it is clear now that Gondwana also held a high phylogenetic diversity of Early Jurassic pterosaurs, with the Cañadón Asfalto Formation alone now exhibiting evidence for at least three distinct taxa. This further highlights our still-lacking knowledge of Jurassic pterosaur faunas from Gondwana, and it is evident that, pending more field sampling and pterosaur fossil recovery, the inherent potential is present for the Southern Hemisphere to perhaps one day match the abundance of the Northern Hemisphere.

## Data Availability

All phylogenetic matrices used are available in the electronic supplementary material [98], as well as at https://morphobank.org/permalink/?P4589. CT scan data are made available at https://www.morphosource.org/concern/media/000649886?locale=en.
